# Quantitative proteome-level analysis of paulownia witches’ broom disease with methyl methane sulfonate assistance reveals diverse metabolic changes during the infection and recovery processes

**DOI:** 10.7717/peerj.3495

**Published:** 2017-07-03

**Authors:** Zhe Wang, Wenshan Liu, Guoqiang Fan, Xiaoqiao Zhai, Zhenli Zhao, Yanpeng Dong, Minjie Deng, Yabing Cao

**Affiliations:** 1 Institute of Paulownia, Henan Agricultural University, Zhengzhou, China; 2 College of Forestry, Henan Agricultural University, Zhengzhou, China; 3 Forestry Academy of Henan, Zhengzhou, China

**Keywords:** Proteome, Differentially abundant proteins, Phytoplasmas, Paulownia witches’ broom, iTRAQ

## Abstract

Paulownia witches’ broom (PaWB) disease caused by phytoplasma is a fatal disease that leads to considerable economic losses. Although there are a few reports describing studies of PaWB pathogenesis, the molecular mechanisms underlying phytoplasma pathogenicity in Paulownia trees remain uncharacterized. In this study, after building a transcriptome database containing 67,177 sequences, we used isobaric tags for relative and absolute quantification (iTRAQ) to quantify and analyze the proteome-level changes among healthy *P. fortunei* (PF), PaWB-infected *P. fortunei* (PFI), and PaWB-infected *P. fortunei* treated with 20 mg L^−1^ or 60 mg L^−1^ methyl methane sulfonate (MMS) (PFI-20 and PFI-60, respectively). A total of 2,358 proteins were identified. We investigated the proteins profiles in PF *vs*. PFI (infected process) and PFI-20 *vs*. PFI-60 (recovered process), and further found that many of the MMS-response proteins mapped to “photosynthesis” and “ribosome” pathways. Based on our comparison scheme, 36 PaWB-related proteins were revealed. Among them, 32 proteins were classified into three functional groups: (1) carbohydrate and energy metabolism, (2) protein synthesis and degradation, and (3) stress resistance. We then investigated the PaWB-related proteins involved in the infected and recovered processes, and discovered that carbohydrate and energy metabolism was inhibited, and protein synthesis and degradation decreased, as the plant responded to PaWB. Our observations may be useful for characterizing the proteome-level changes that occur at different stages of PaWB disease. The data generated in this study may serve as a valuable resource for elucidating the pathogenesis of PaWB disease during phytoplasma infection and recovery stages.

## Introduction

Witches’ broom disease is caused by a plant phytoplasma, which spread by sap-sucking insect vectors (Lee, Davis & Gundersen-Rindal, 2000). It has been found in many plant species (Maejima, Oshima & Namba, 2014), including Paulownia (Liu et al., 2013). Phytoplasma-infected plants usually contain witches’ brooms, and appear yellow and stunted (Hogenhout et al., 2008). Witches’ broom disease is known to considerably decrease forest productivity. Although the disease has been thoroughly investigated over the past few decades, its pathogenesis remains largely uncharacterized. Several studies revealed that in infected plants, the phytoplasmas inhibit photosynthesis, carbohydrate metabolism, and hormone balance, as well as induce the development of disease symptoms (Hogenhout et al., 2008; Kube et al., 2012). Additionally, some studies that focused on insect vectors and their interactions with phytoplasmas have found that phytoplasmas cause the insect vectors to lay more eggs, and that the major antigenic membrane proteins influence the transmission of phytoplasmas (Beanland et al., 2000; Rashidi et al., 2015). Because phytoplasmas lack many endogenous metabolic genes, they survive on the metabolic compounds obtained from their hosts. This characteristic may contribute to the difficulties encountered in cultivating phytoplasmas *in vitro*, which have limit the study of witches’ broom disease. The development of ‘omics’ technologies has led to the publication of the genomes of five phytoplasmas. Furthermore, many genes (Liu et al., 2013; Luge et al., 2014; Mardi et al., 2015; Mou et al., 2013), miRNAs (Ehya et al., 2013; Fan et al., 2015a; Gai et al., 2014), and some virulence factors (Minato et al., 2014; Tan et al., 2016) related to witches’ broom disease have been identified. These results may be useful for characterizing host—pathogen relationships and the mechanisms regulating the pathogenesis of witches’ broom disease.

*Paulownia fortunei* is a fast-growing deciduous hardwood species with adaptive capacity to exist in diverse climates and soil conditions. It is particularly promising for afforestation and ecological improvement. *P. fortunei* has a global distribution, but is not grown in Antarctica (Hall, 2008). Paulownia trees are widely used during forestation, and are also useful for producing furniture and laminated structural beams (Yadav et al., 2013). Additionally, Paulownia trees have been grown as an energy crop and as a potential source of traditional Chinese medicine (Ji et al., 2015; López et al., 2012). Although Paulownia trees are economically and ecologically valuable, their production declines significantly after PaWB infections. We recently observed that treating PaWB-infected Paulownia seedlings with methyl methane sulfonate (MMS) resulted in morphologically healthy plants in which the phytoplasmas had been eliminated. Additionally, MMS treatment has been used to mimic the disease recovery process that enables Paulownia plants to overcome infections by the PaWB-causing phytoplasma (Cao et al., 2014; Liu et al., 2013). Some studies used high-throughput sequencing to identify genes and miRNAs related to PaWB (Fan et al., 2015a; Fan et al., 2015b; Fan et al., 2014; Fan et al., 2016; Fan et al., 2015c; Liu et al., 2013). However, the molecular mechanisms underlying PaWB disease were poorly characterized. Proteins are responsible for mediating biological activities; therefore, combining transcriptomics with proteomics may provide more complete and useful information (Oliver, Nikolau & Wurtele, 2002). Previously, we applied two-dimensional gel electrophoresis (2-DE) to investigate proteome-level changes induced by PaWB disease. We identified a PaWB-related protein, chloroplast molecular chaperone, and characterized it with pI6.8, 24 kD properties (Fan et al., 2003).

A limitation of 2-DE is that low-abundance proteins are generally under-represented. Alternatively, iTRAQ (isobaric tags for relative and absolute quantification) is recognized as a highly sensitive method for revealing changes in protein abundance (Ma et al., 2016). In this study, we applied iTRAQ to generate protein profiles for *P. fortunei* seedlings infected by or recovering from PaWB disease. Our data will help to characterize PaWB disease. Furthermore, the identified PaWB-related proteins may be relevant for developing disease-resistant Paulownia varieties in plant breeding programs.

## Materials & Methods

### Plant materials

All the biological material used in this study were obtained from the Institute of Paulownia, Henan Agricultural University, China. The following five groups of *P. fortunei* seedlings were included: healthy *P. fortunei* (PF), PaWB-infected *P. fortunei* (PFI), and PaWB-infected *P. fortunei* treated with 20 mg L^−1^, 60 mg L^−1^, or 100 mg L^−1^ MMS (PFI-20, PFI-60, and PFI-100, respectively). The cultivation and treatment procedures were as described by Fan et al. (2014). The terminal buds from three individual plants were combined to form one biological replicate, and at least three biological replicates were used for each treatment.

### Sequence assembly

The unigenes used in this study were from the PF, PFI, PFI-20, and PFI-60 transcriptome libraries developed in previous studies (Fan et al., 2014; Fan et al., 2015c). The assembly, bioinformatics analysis, and functional annotations were completed as described in two previous studies (Fan et al., 2014; Fan et al., 2015c). Briefly, after sequences were filtered, we obtained 120,963 unigenes (Table S1). The sequencing data have been submitted to the Short Reads Archive (accession number SRP067302). The unigenes were aligned against NCBI’s non-redundant protein database (Nr), Swiss-Prot, Gene Ontology (GO), Kyoto Encyclopedia of Genes and Genomes (KEGG), and Clusters of Orthologous Groups of proteins (COG) databases, and a total of 83,179 protein-coding sequences (CDSs) were predicted: 82,221 CDSs were inferred from the BLASTX hits and 958 were assigned using ESTScan. After removing redundant sequences, we obtained a transcriptome database that contained 67,177 unique unigene sequences.

### Protein preparation

Proteins were extracted from four *P. fortunei* accessions (PF, PFI, PFI-20 and PFI-60) using a previously described procedure (Tian et al., 2013). Two replicates for each accession. The proteins in the supernatant were kept at −80 °C until used in the subsequent analyses.

### Proteome analysis by iTRAQ

The extracted proteins were digested and labeled as described by Qiao et al. (2012). The labeled proteins were extracted from the PF, PFI, PFI-20 and PFI-60. The labeled peptide mixtures were pooled for strong cation-exchange (SCX) chromatography and then dried by vacuum centrifugation. The SCX chromatography was completed as described by Dong et al. (2016) using an LC-20AB HPLC pump system (Shimadzu, Kyoto, Japan). The fractionated samples were analyzed by liquid chromatography—electrospray ionization tandem mass spectrometry (LC−ESI−MS/MS) based on a Triple TOF 5600 system (AB SCIEX, Framingham, MA, USA), as previously described (Qiao et al., 2012).

### Database search and quantification

Raw data files were converted into MGF files using the Proteome Discoverer software to generate a peak list (Lin et al., 2013). Proteins were identified and quantified using the Mascot 2.3.02 search engine (Matrix Science, London, United Kingdom) and compared against the transcriptome database of 67,177 unigene sequences. The quantitative protein ratios were weighted and normalized according to the median ratio method of Mascot. Proteins with *p*-values <0.05, and fold changes >1.2 were considered as differentially abundant proteins (DAPs) (Chu et al., 2015; Dong et al., 2016).

### Analysis of PaWB-related DAPs

To identify DAPs related to PaWB, we made comparisons among the four samples using a previously reported method (Cao et al., 2014). Functional analyses of the identified proteins were conducted using the GO, COG, and KEGG databases.

### RNA preparation and quantitative RT-PCR

Total RNA was extracted from the samples used for the iTRAQ analysis. The RNA extraction and qRT-PCR procedures were conducted as previously described (Fan et al., 2014; Fan et al., 2015c). We randomly selected DAPS to investigate their expression at the transcript level. Gene-specific primers were designed using Beacon Designer (version 7.7) (Premier Biosoft International, Palo Alto, CA, USA), and their efficiencies were checked according to the standard curve method. The primer specificities were assessed with melting curves after all the qRT-PCR runs. The sequences of the forward and reverse primers used in this study are provided in Table S2. Statistical analysis was performed using SPASS 19.0 (SPASS, Inc., Chicago, IL, USA). A Student’s t test was used to detect differences at a significance level of *p* = 0.05.

## Results

### Morphological changes of differently treated seedlings

**Figure 1:**
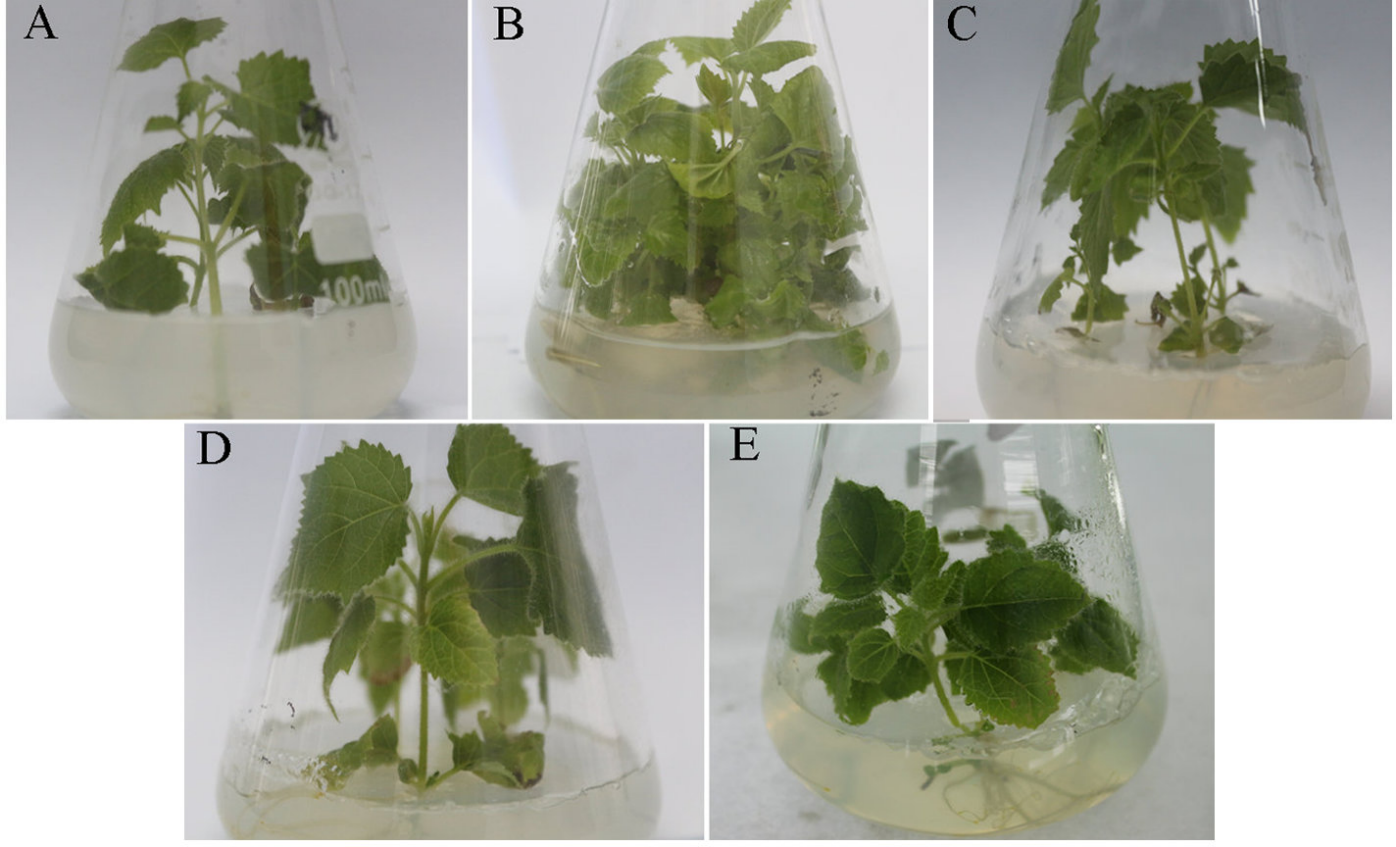
Change of the morphology of Paulownia seedlings. (A) The healthy wild-type sample of *P. fortunei,* (B) the sample of phytoplasma infected PF, (C) the sample of 20 mg L^−1^ MMS treated PFI, (D) the sample of 60 mg L^−1^ MMS treated PFI, (E) the sample of 100 mg L^−1^ MMS treated PFI.

The PFI seedlings exhibited a witches’ broom phenotype (e.g., yellowing and relatively small leaves, short internodes, and phyllody) (Fig. 1B). The PFI-20 seedlings exhibited an asymptomatic morphology (Fig. 1C), while the PFI-60 seedlings returned to being morphologically healthy (Fig. 1D). The PFI-100 samples appeared healthy, but exhibited delayed growth (Fig. 1E). The phytoplasma was detected in the PFI and PFI-20 samples by nested-PCR (Cao et al., 2014), but not in the PF, PFI-60, and PFI-100 seedlings (Fig. S1 and Table S2). To reduce the influence of MMS on plant growth, we eliminated PFI-100 and chose PFI-20 and PFI-60 as the MMS-treated materials for studying the PaWB disease recovery process.

### Proteome characterization

A total of 458,154 spectra were generated from the iTRAQ-based analysis of the total proteins extracted from the PF, PFI, PFI-20, and PFI-60 seedlings. After filtering the data to exclude low-scoring spectra, 22,544 unique spectra that matched specific peptides were obtained. Additionally, 2,358 proteins were finally identified (Table S3), and the proteomic results were reliable (Fig. S2).

To predict the functions of the 2,358 proteins, they were annotated by searches against the COG, GO, and KEGG databases and were assigned to 23 COG categories (Fig. S3), 54 GO groups (Fig. S4), 121 KEGG pathways (Table S4). The KEGG analysis results indicated that most of the mapped proteins may affect Paulownia metabolic activities.

### DAPs involved in the PaWB infection and recovery processes

Proteins with relative abundance fold changes >1.2 (*p* < 0.05) were defined as DAPs (Chu et al., 2015; Dong et al., 2016). In the PaWB infection process (PF *vs.* PFI), we found 233 DAPs that may be involved; 113 exhibited increased abundance and 120 exhibited decreased abundance in PFI compared with PF (Table S5). The 233 DAPs were assigned to 83 KEGG pathways, including the highly enriched “ribosome”, “photosynthesis”, “carbon fixation in photosynthetic organisms”, “glyoxylate and dicarboxylate metabolism”, and “glycolysis/gluconeogenesis” pathways (Table S6). Under the three main GO categories, biological process, cellular component, and molecular function, 139, 65, and 16 GO terms were significantly enriched, respectively (Table S7 and Fig. S5).

In the recovery process (PFI-20 *vs.* PFI-60), we found 129 DAPs that may be involved; 67 showed higher abundance and 62 showed lower abundance in PFI-60 compared with PFI-20 (Table S5). The 129 DAPs were mapped to 47 KEGG pathways, and two metabolic pathways, “photosynthesis” and “ribosome”, were found to be significantly enriched (Table S8). Photosynthesis-related proteins were identified previously in the recovered ‘Barbera’ grapevines (Margaria, Abba & Palmano, 2013). In the GO analyses, 152 biological process, 57 cellular component, and 57 molecular function GO terms were significantly enriched (Table S9 and Fig. S5).

A smaller number of DAPs were associated with the recovery process compared with the infection process, so logically, the DAPs in the recovery process would map to fewer KEGG pathways and GO terms. However, under molecular function, the significantly enriched GO terms associated with the recovery process were more than those associated with the infection process. The difference was mainly in GO terms associated with “binding”; in PF *vs.* PFI, only two “binding” GO terms were enriched, while, in PFI-20 *vs.* PFI-60, 23 “binding” GO terms were enriched. In addition, the “photosynthesis” and “ribosome” metabolic pathways were enriched in both the infection and recovery processes, suggesting these pathways were active in both processes.

To determine the effect of MMS in the control of PaWB disease, we compared PFI *vs.* PFI-20 and PFI-20 *vs.* PFI-60, and identified 155 and 129 DAPs, respectively (Table S5). Forty of these DAPs were common in the two comparison (Table S10), suggesting they may be MMS-related. Many of these 40 DAPs were mapped to “photosynthesis” and “ribosome” metabolic pathways. The roles of these two metabolic pathways should be the subject of an in-depth study in the future.

### DAPs associated with PaWB

**Figure 2:**
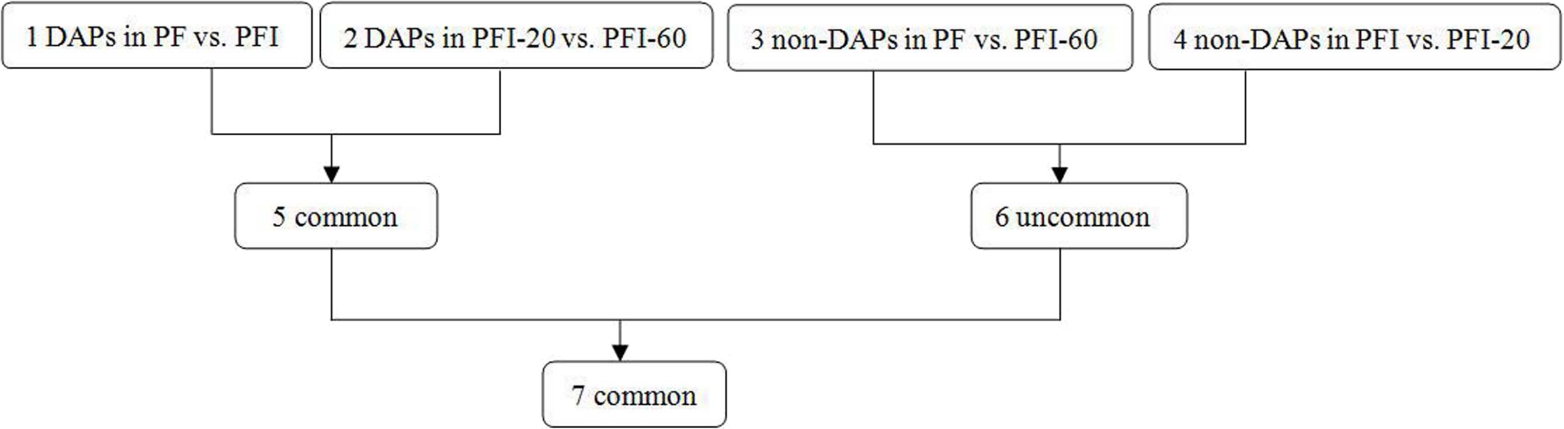
Comparison schemes of the four samples. PF represents the healthy wild-type sample of *P. fortunei,* PFI represents the sample of phytoplasma infected PF. PFI-20 represents the sample of 20 mg L^−1^ MMS treated PFI, PFI-60 represents the sample of 60 mg L^−1^ MMS treated PFI.

**Figure 3:**
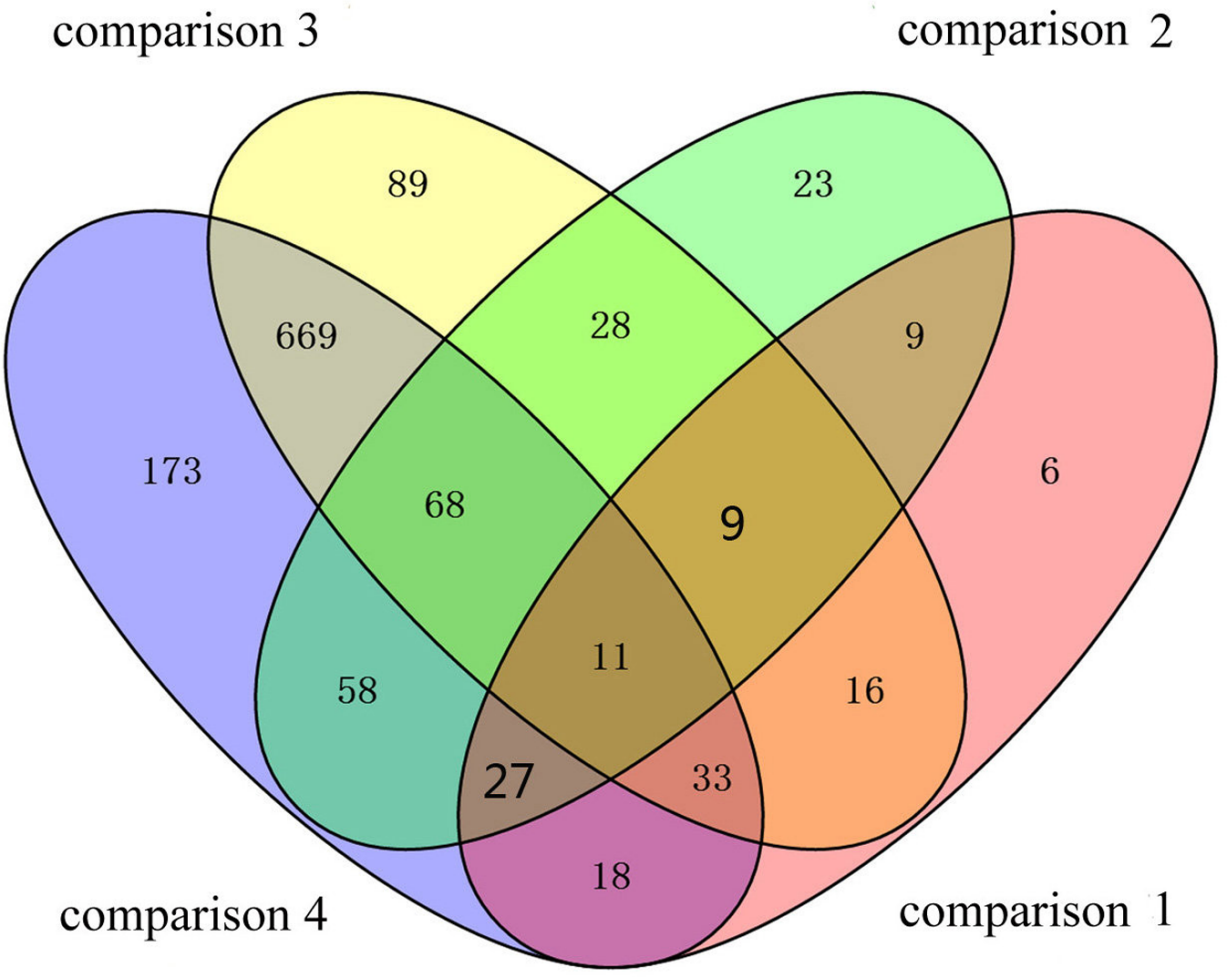
Details of the comparison schemes. Comparison 1: DAPs in PFI-20 *vs.* PFI-60, comparison 2: DAPs in PF *vs.* PFI, comparison 3: non-DAPs in PF *vs.* PFI-60, comparison 4: non-DAPs in PFI *vs.* PFI-20.

To identify the DAPs associated with PaWB, we used a comparison scheme from a previous study as follows (Cao et al., 2014) (Fig. 2). The 233 DAPs in PF *vs*. PFI (comparison 1) were related to PaWB and other factors like difference of plant growth and development (DPGD), and the 129 DAPs in PFI-20 *vs.* PFI-60 (comparison 2) were related to PaWB, DPGD, and/or differences in the MMS treatments. We also identified 923 non-DAPs in PF *vs*. PFI-60 (comparison 3), which were related to DPGD, and 1057 non-DAPs in PFI *vs*. PFI-20 (comparison 4), which were related to PaWB and DPGD. Additionally, we detected 56 common DAPs between comparisons 1 and 2 (comparison 5), which were related to PaWB and DPGD; 418 specific proteins between comparisons 3 and 4 (comparison 6), which were judged as related to PaWB; and 36 common DAPs from comparison 5 and 6, which may be related to PaWB (Fig. 3, Table 1 and Table S5). The DAPs associated with PaWB were assigned to 35 GO categories (Table S11). The most represented GO terms were also the most represented among all the proteins detected, indicating their functions were important in Paulownia. The PaWB-associated DAPs were assigned to seven COG categories (Table S12) and 16 KEGG pathways (Table S13). The most represented pathways were metabolic pathways (11 DAPs), photosynthesis (6 DAPs), ribosome (4 DAPs), and biosynthesis of secondary metabolites (3 DAPs); the others pathways mostly contained two/one DAPs. This result indicates that photosynthesis and ribosome may be important in the plant’s response to PaWB.

**Table 1:** Annotations of the 36 PaWB-related proteins. Protein accession data is available via ProteomeXchange under project identifier PXD006731.

Protein accession	Annotations	Species	Reference
**Carbohydrate and energy metabolism**			
CL11786.Contig1_All	Light-harvesting complex I chlorophyll a/b binding protein 3	Arabidopsis, Physcomitrella	Alboresi et al. (2008), Mozzo et al. (2006)
CL5304.Contig1_All	Light-harvesting complex II chlorophyll a/b binding protein 5	Arabidopsis, tobacco	Chen et al. (2016), Wehner, Grasses & Jahns (2006)
CL832.Contig4_All	Photosystem I P700 chlorophyll a apoprotein A1	Arabidopsis, maize	Fish & Bogorad (1986), Muranaka et al. (2012)
CL11274.Contig1_All	Cytochrome b6f Rieske iron-sulfur subunit	Arabidopsis, Watermelon	Munekage et al. (2001), Sanda et al. (2011)
CL1718.Contig1_All	Cytochrome b6	Arabidopsis, Pea	Kroliczewski et al. (2016), Stoppel et al. (2011)
CL1900.Contig1_All	Photosystem II oxygen-evolving enhancer protein 1	Arabidopsis, tobacco	Allahverdiyeva et al. (2013), Ifuku et al. (2004)
Unigene11415_All	Photosystem II 10 kDa protein	Arabidopsis, tomato	John et al. (1997), Liu, Frankel & Bricker (2009)
Unigene7821_All	Ribulose bisphosphate carboxylase (small chain) family protein	Arabidopsis, tomato	Manzara, Carrasco & Gruissem (1993), Zhan et al. (2014)
Unigene8915_All	Photosystem II Psb27 protein	Arabidopsis, cyanobacterial	Grasse et al. (2011), Hou et al. (2015)
CL8613.Contig1_All	Thioredoxin Mtype 4	Arabidopsis, tobacco	Courteille et al. (2013), Okegawa & Motohashi (2015)
Unigene12498_All	Cytosolic pyruvate kinase	Arabidopsis, potato	Andre et al. (2007), Oliver et al. (2008)
**Protein synthesis and degradation**			
CL896.Contig2_All	HopW1-1-Interacting protein 1	Arabidopsis, tobacco	Fremont et al. (2013), Vinatzer et al. (2006)
CL12527.Contig2_All	Glycine decarboxylase complex H protein	Arabidopsis, poplar	Srinivasan & Oliver (1995), Wang, Harding & Tsai (2004)
Unigene31090_All	Ribosomal protein L5 B	Arabidopsis, alfalfa	Asemota et al. (1994), Van Minnebruggen et al. (2010)
CL13424.Contig1_All	Ribosomal protein L6	Bacillus, coli	Gulati et al. (2014), Shigeno, Uchiumi & Nomura (2016)
CL5472.Contig1_All	Ribosomal protein S20	Arabidopsis	Fares, Rossignol & Peltier (2011)
CL8143.Contig1_All	Plastid-specific ribosomal protein 4	Arabidopsis, spinach	Tiller et al. (2012), Yamaguchi & Subramanian (2003)
CL5429.Contig1_All	Ribosomal protein L12	Arabidopsis, tobacco	Nagaraj et al. (2015)
Unigene31051_All	20S proteasome beta subunits D1	Arabidopsis, rice	Fu et al. (1998), Sassa et al. (2000)
**Stresses resistances**			
CL4968.Contig5_All	Heat Shock Protein 90	Arabidopsis, tobacco	Shibata, Kawakita & Takemoto (2011), Takahashi et al. (2003)
CL2738.Contig1_All	Stress-inducible protein	Arabidopsis, rice	Prasad, Goel & Krishna (2010)
CL4391.Contig1_All	Co-chaperone GrpE family protein	Arabidopsis tobacco	Kim et al. (2010), Padidam et al. (1999)
Unigene5139_All	GroES-like family protein	Pea, spinach	Bertsch et al. (1992)
Unigene9539_All	Rotamase cyclophilin 5	Arabidopsis, peanut	Kumar & Kirti (2011), Saito et al. (1999)
CL4998.Contig1_All			
CL7006.Contig1_All	FK506- and rapamycin-binding protein 15 KD-2	Arabidopsis, fava bean	Luan et al. (1996), Mokryakova et al. (2014)
CL4603.Contig2_All	Ferredoxin/thioredoxin reductase	Arabidopsis, maize	Iwadate et al. (1996), Keryer et al. (2004)
Unigene20234_All	Peroxiredoxin Q	Arabidopsis, poplar	Lamkemeyer et al. (2006), Rouhier et al. (2004)
CL2226.Contig1_All	Clathrin light chain 2	Arabidopsis, tobacco	Van Damme et al. (2011), Fujimoto et al. (2007)
CL4105.Contig3_All	LysM domain GPI-anchored protein 1 precursor	Arabidopsis	Willmann et al. (2011)
CL9243.Contig2_All	Nascent polypeptide associated complex alpha chain	Arabidopsis, soybean	Jiang et al. (2007), Zhang et al. (2011)
Unigene2121_All	MLP-like protein 423	Arabidopsis, apricot	Jr et al. (2016), Rubio et al. (2015)
Unknown			
CL5710.Contig2_All			
Unigene1547_All			
Unigene30260_All			
Unigene8870_All			

We used BLASTX alignments to confirm the functional annotations of the 36 PaWB-related proteins (Table 1), and classified them into three groups: (1) carbohydrate and energy metabolism, (2) protein synthesis and degradation, and (3) stress resistance.

#### Carbohydrate and energy metabolism

Photosynthesis can be affected by phytoplasma infections, as has been documented previously (Hren et al., 2009; Nejat et al., 2015). After phytoplasma infection, many plants exhibited impaired photosynthesis and accumulated carbohydrates (Christensen et al., 2005; Hren et al., 2009; Liu et al., 2013; Mou et al., 2013; Nejat et al., 2015). In our study, 11 proteins related to carbohydrate and energy metabolism were identified. Nine proteins showed lower abundance in PFI compared with PF, including light-harvesting complex I chlorophyll a/b binding protein 3 (Lhca3), light-harvesting complex II chlorophyll a/b binding protein 5 (Lhcb5), photosystem I P700 chlorophyll a apoprotein A1 (PsaA), cytochrome *b*_6_*f* Rieske iron-sulfur subunit (PetC), photosystem II oxygen-evolving enhancer protein (PsbP1), photosystem II 10 kDa protein (PsbR), ribulose-bisphosphate carboxylase small chain family protein (rbcS), thioredoxinMtype 4 (TrxM4) ,cytosolic pyruvate kinase (PKc), while two proteins, cytochrome b6 (PetB) and photosystem II Psb27 protein (Psb27), showed higher abundance.

Callose deposition is a common phenomenon that has been demonstrated in the sieve elements of *Catharanthus roseus* and *Euphorbia pulcherrima* infected with phytoplasmas and is associated with the accumulation of carbohydrates (Christensen et al., 2004). The accumulated free hexoses can repress the synthesis of chlorophyll a/b-binding proteins (Sheen, 1990). Lhca3 and Lhcb5 are chlorophyll a/b-binding proteins that coordinate the antenna pigments in the light-harvesting complex of photosystems PSI and PSII (Alboresi et al., 2008). They capture solar energy for the primary light reactions of photosynthesis (Wehner, Grasses & Jahns, 2006). The decreased abundance of Lhca3 and Lhcb5 in PFI in our study may influence the light-harvesting rate, and induce the transfer of electrons (Chen et al., 2016; Mozzo et al., 2006). In a proteomic analysis of pear plants, the abundance of Lhca3 decreased after phytoplasma infections (Del Prete et al., 2011).

In Chardonnay grapes infected by ‘Bois noir’ phytoplasma, serious inhibition of the whole photosynthetic chain and PSI activity as well as Calvin-cycle enzyme transcription was observed (Albertazzi et al., 2009). PsaA binds to the electron donor P700, and functions as the electron acceptor in the PSI electron transfer chain. Thus, PsaA is associated with PSI activity (Fish & Bogorad, 1986; Muranaka et al., 2012). The Cyt *b*_6_*f* complex is present in the thylakoid membrane, and transports electrons from PSII to PSI (Kroliczewski et al., 2016; Sanda et al., 2011). PetB and PetC along with other subunits influence the formation and stability of the Cyt *b*_6_*f* complex (Stoppel et al., 2011). In *Arabidopsis thaliana*, a mutation in *petc* was shown to influence electron transport (Munekage et al., 2001). The oxygen-evolving complex of eukaryotic PSII consists of four extrinsic subunits, PsbO, PsbP, PsbQ, and PsbR, which participate in the water-splitting reaction (Ifuku et al., 2004). PsbP and PsbR are involved in oxygen evolution and PSII electron transport (Allahverdiyeva et al., 2013; Liu, Frankel & Bricker, 2009). Mou et al. (2013) reported that the genes encoding PsbP1 and PsbR were down-regulated after phytoplasma infections. In our study, the expression of the unigene encoding PsbP1 was verified by qRT-PCR. PsbR is similar to a pathogenesis-related tomato protein (John et al., 1997). Thioredoxins regulate the activities of various chloroplast proteins in a light-dependent manner (Okegawa & Motohashi, 2015), and, in *A. thaliana*, TrxM4 controls alternative photosynthetic electron pathways (Courteille et al., 2013). In our study, some of the proteins that showed decreased abundance in PFI, namely PsaA, PetC, PsbP1, PsbR, and Trxm4, are related to electron transport, implying that PaWB infection may influence the electron transfer chain in Paulownia.

In infected grape, the large subunit of Rubisco and Rubisco activase were inhibited (Hren et al., 2009). The rbcS protein plays an important role in the Calvin cycle, and the lower abundance of rbcS in FPI observed in this study may indicate that photosynthesis has been affected (Manzara, Carrasco & Gruissem, 1993; Zhan et al., 2014). This protein also has been detected in phytoplasma-infected pear trees (Del Prete et al., 2011). The PKc enzyme catalyzes the ADP-dependent conversion of phosphoenolpyruvate to pyruvate while producing ATP in the carbon fixation pathways, and is associated with growth and respiration (Andre et al., 2007; Oliver et al., 2008). The decreased abundance of PKc and rbcS in PFI suggested that the carbon fixation may be influenced in response to phytoplasma infection. Thus, upon being infected, photosynthesis was down-regulated in the PFI seedlings because of the inhibition of carbohydrate and energy metabolism.

Psb27 influences photosystem biogenesis and recovery from photodamage (Hou et al., 2015), which is involved in repairing PSI (Grasse et al., 2011). Under stressful conditions, Psb27 is highly accumulated in cyanobacteria to ensure survival. Psb27 can recover damaged photosystems, suggesting its abundance increased in PFI to satisfy the need of Paulownia. The PaWB-related proteins mentioned above have various functions that influence photosynthesis. Thus, after phytoplasma infections, the lower abundance of these DAPs may have influenced the photosynthetic activities of the infected seedlings, ultimately affecting forest productivity.

#### Protein synthesis and degradation

Phytoplasmas lack an amino acid synthesis pathway and rely on the host to survive. Thus, phytoplasma infections influence the protein synthesis and degradation of the host to some extent. In this study, we identified two amino acid metabolism-related DAPs, HopW1-1-Interacting protein 1(WIN1) and glycine decarboxylase complex H protein (GDC-H), and six protein metabolism-related DAPs, ribosomal protein L5B (RPL5B), ribosomal protein L6 (RPL6), ribosomal protein L12 (RPL12), ribosomal protein S20 (RPS20), plastid-specific ribosomal protein 4 (PSRP4) and 20S proteasome subunit beta D1 (PBD1).

The WIN1 protein is a putative acetylornithine amino transferase (argD), which catalyzes the fourth step of the arginine biosynthesis pathway (Fremont et al., 2013). Arginine is an essential amino acid for protein synthesis, and it is also a nitrogen storage compound (Fremont et al., 2013). After phytoplasma infections, arginine production is induced by accumulated ArgD, indicating that protein synthesis might be stimulated by PaWB. ArgD is also an important endogenous substrate for the synthesis of NO, which acts as a signaling molecule in different plant tissues and during pathogen-induced hypersensitive responses (Delledonne et al., 2001). Furthermore, WIN1 interacts with the *Pseudomonas syringae* defense-inducing effector HopW1-1 to modulate plant defenses (Vinatzer et al., 2006). The glycine decarboxylase complex (GDC) contributes to the generation of one-carbon units for the biosynthesis of primary and secondary metabolites (Srinivasan & Oliver, 1995). The GDC-H protein affects the degradation of glycine and is associated with one-carbon metabolism (Wang, Harding & Tsai, 2004). The decreased abundance of GDC-H protein detected in PFI may inhibit the degradation of glycine and disturb the primary and secondary metabolites in infected Paulownia. The phytoplasmas survive on the amino acids supplied by the infected host. In the PFI seedlings, we found that proteins related to amino acid synthesis showed higher abundance, and proteins related to amino acid degradation showed lower abundance, might result in the amino acid content increasing.

The eukaryotic ribosome is a complex structure composed of four rRNAs and about 80 ribosomal proteins. In our study, five ribosomal proteins were differentially abundant; four exhibited decreased abundance, suggesting the corresponding protein synthesis was insufficient. Ribosomal proteins have other functions, apart from their roles related to protein synthesis. For example, RPL5B plays a key role in cell expansion during organ growth (Asemota et al., 1994; Van Minnebruggen et al., 2010). Some reports have suggested that RPL6 interacts directly with GTPase translation factors, and influences ribosome maturation (Gulati et al., 2014; Shigeno, Uchiumi & Nomura, 2016). Possible interactions between RPS20 and the stress-induced elongation factor LOS1 have also been reported (Fares, Rossignol & Peltier, 2011). The plastid-specific ribosomal proteins (PSRPs) are accessory proteins involved in translational regulation (Yamaguchi & Subramanian, 2003). The down-regulation of PSRP4 leads to the development of pale-green leaves and severely retarded growth (Tiller et al., 2012), which is similar to some PaWB symptoms. The lower abundance of PSRP4 in PFI may be related to the observed morphological changes. RPL12 is a ribosomal protein that contributes to non-host disease resistance against bacterial pathogens (Nagaraj et al., 2015). Our results revealed that RPL12 abundance increased in PFI compared with PF, possibly because of its role in responding to phytoplasma infection. Furthermore, changes in the abundance of ribosomal proteins suggest that the Paulownia translation machinery was altered by the PaWB phytoplasma. Upon phytoplasma infection, four ribosomal proteins (RPL5B, RPL6, RPS20, PSRP4) decreased in abundance, which would inhibit protein synthesis and increase the amount of free amino acids for the phytoplasma.

Proteolytic enzymes are essential for the degradation of damaged and misfolded proteins during the plant life cycle (Fu et al., 1998; Sassa et al., 2000). In this study, PBD1, which accumulated in PFI after phytoplasma infection, was identified as associated with protein degradation. Its accumulation might help to increase the pool of free amino acids for phytoplasma nutrition. Similar results were obtained for infected mulberry and lime (Ji et al., 2009; Monavarfeshani et al., 2013). Thus, the changes in abundance of these proteins might benefit the PaWB-causing phytoplasma. The elevated levels of amino acid synthesis, enzymes, and ribosomal proteins may reflect the increased need for amino acids and the decreased protein biosynthetic capacity of infected Paulownia trees.

#### Stress resistance

Because of the stress caused by microbial pathogens, plants have evolved a response system that can efficiently detect and ward off potential pathogens. During these responses, the production of stress-related proteins may be induced. In this study, we detected a number stress-related proteins among the DAPS, including Heat Shock Protein 90 (HSP90), stress-inducible protein(SIP), Co-chaperone GrpE family protein (Co-GrpE), GroES-like family protein (GroES-L), two rotamasecyclophilin 5 (ROC5), FK506- and rapamycin-binding protein 15 KD-2 (FKBP15-2), Ferredoxin/thioredoxin reductase (FTR), peroxiredoxin Q (PrxQ), clathrin light chain 2 (CLC2), LysM domain GPI-anchored protein 1 precursor (LYM1), nascent polypeptide associated complex alpha chain (*α*-NAC) and MLP-like protein 423 (MLP-423).

Many molecular chaperones are produced in response to environmental stresses, including heat shock proteins (HSPs) such as HSP90, HSP70, and HSP40. HSP90 is one of the most conserved HSPs, and is involved in signal transduction, protein trafficking, and innate and adaptive immunity (Shibata, Kawakita & Takemoto, 2011). In *A. thaliana* and tobacco plants, HSP90 contributes to disease resistance (Shibata, Kawakita & Takemoto, 2011; Takahashi et al., 2003). SIP is one of the carboxylate clamp-type tetratricopeptide repeat proteins, and it also acts as a co-chaperone of Hsp90/Hsp70 (Prasad, Goel & Krishna, 2010). In *A. thaliana*, immunity to bacterial infections requires LysM domain proteins that can recognize GlcNAc-containing glycans (Willmann et al., 2011). LYM1 production is reportedly induced by phytoplasma infections in lime (Monavarfeshani et al., 2013), possibly because LYM1 can recognize some of the phytoplasma “glycans”. HSP90 and LysM could play important roles in the immune system of Paulownia. After infected, they showed higher abundance as a response.

The GroES and GrpE proteins bind to GroEL (HSP60) and DnaK (HSP70), respectively, in the presence of ATP (Bertsch et al., 1992; Padidam et al., 1999). DnaK cooperates with GrpE to ensure proteins are folded and assembled correctly (Kim et al., 2010). The folding cage generated by GroEL/GroES has two functions related to protein folding: it prevents the aggregation of the substrate protein and accelerates the folding process (Brinker et al., 2001). In our study, the increased abundance of GroES-L in PFI may help maintain normal protein folding activities, even under PaWB-induced stress conditions. Additionally, the Hsp70-type (*dnaK*,*grpE*) and Hsp60-type (*groEL*, *groES*) chaperone systems have been identified in phytoplasmas (Kube et al., 2012). Co-GrpE and GroES-L are members of the chaperone systems that have been found in phytoplasma, and their increased abundance may be resulted from the phytoplasma infection.

The cyclophilins (CyPs), FKBPs, and parvulins exhibit peptidyl–prolylcis–trans isomerase (PPIase) activity, and may be important for mRNA processing, signal transductions, and responses to pathogens (Kumar & Kirti, 2011). PPIases of ROC5 and FKBP15-2 are susceptibility factors in plant–pathogen interactions (Milli et al., 2012), and their lower abundance might be caused by phytoplasmas. ROC5 is produced in vascular tissues and flowers, and regulates *Pseudomonas syringae* infections (Kumar & Kirti, 2011; Saito et al., 1999). Our results indicate that ROC5 may be associated with phytoplasma infection of Paulownia trees. FKBP15 is encoded by a small gene family in higher plants and is responsive to stresses (Luan et al., 1996). In *A. thaliana*, the *fkbp15-2* mutant exhibits greater susceptibility to pathogens (Mokryakova et al., 2014). The decreased abundance of FKBP15-2 in PFI may have enabled the phytoplasma to survive in the Paulownia seedlings. Furthermore, an FKBP-type immunophilin is required for the accumulation of the PSII supercomplex (Lima et al., 2006), and the lower abundance of FKBP15-2 in PFI might be correlated with an overall decline in photosynthetic activities.

FTR is the key enzyme of a light-dependent redox regulatory system that controls enzyme activities in oxygenic photosynthetic cells (Iwadate et al., 1996). FTR catalyzes the reversible transfer of electrons between the one-electron carrier ferredoxin and a single molecule of Trx (Keryer et al., 2004). The Prx family includes ubiquitous Trx or glutaredoxin-dependent peroxidases, which degrade peroxides. PrxQ is one of the four plant Prx subtypes, and participates in general antioxidant defense responses, which protect photosynthetic activities (Lamkemeyer et al., 2006). In poplar, PrxQ production is down-regulated during infections, which is consistent with the results of our study (Rouhier et al., 2004). FTR and PrxQ play an antioxidative function in plants, and their lower abundance in PFI may be evidence for the survival of the phytoplasma in Paulownia.

CLC2 and the adaptin-like protein, TPLATE, influence plant cytokinesis (Van Damme et al., 2011). Clathrin is associated with endocytosis activity, auxin distribution, and transportation (Fujimoto et al., 2007; Kitakura et al., 2011). In this study, the CLC2 level decreased after infections, which may have induced variations in auxin and plant cytokinesis levels. CLC2 is also associated with endocytosis. In PFI, material and transport was disturbed the lower abundance of CLC2 might reflect this phenomenon. It has been demonstrated that *α*-NAC helps to correctly orient nascent polypeptides at ribosomes with directional factors such as transcriptional coactivators, and may be induced by biotic and abiotic stresses (Yotov, Moreau & St-Arnaud, 1998). Its decreased abundance had been found in soybean (Zhang et al., 2011) and *A. thaliana* (Jiang et al., 2007), and may lead to an increase in the number of misfolded and dysfunctional proteins (Zhang et al., 2011). The decreased abundance of *α*-NAC could result in proteins being misfolded, and this could act together with the increased abundance of PBD1, which could degrade the abnormal proteins. The MLPs are distantly related to a group of pathogenesis-related proteins (Osmark, Boyle & Brisson, 1998). In apricot, MLP-423 accumulates in infected plants, and is believed to help mediate pathogen resistance (Rubio et al., 2015). In *A. thaliana*, MLP-423, which is regulated by miRNA394, interacts indirectly with the F-Box protein to stimulate the leaf curling responsiveness (Jr et al., 2016). MLP-423 may be involved in the interaction between Paulownia and phytoplasmas. In our study, the observed increased abundance of MLP-423 may be in response to PaWB, and may, therefore, related to the development of PaWB symptoms, such as the yellowing of leaves and the production of abnormally small leaves.

### Correlation between proteins and transcripts

Differentially expressed unigenes (DEUs) were identified in the transcriptome data based on an absolute fold change value of log2 ratio >1 with *p* < 0.001 and a false discovery rate <0.001. In the pairwise comparisons, PF *vs.* PFI, PFI-20 *vs.* PFI-60, PF *vs.* PFI-60, PFI *vs.* PFI-20, we detected 120,472, 119,549, 120,530, and 119,149 unigenes that were expressed at different levels, respectively. However, only 18,636, 4,674, 10,577, and 6,158 unigenes satisfied the criteria to be considered DEUs. We compared the changes in protein abundance with the alterations in transcript levels of corresponding unigenes. If a protein identified quantitatively by iTRAQ had a corresponding unigene that showed transcriptional changes, we considered the protein to be correlated with the transcriptome.

In PF *vs.* PFI, all 2,358 identified proteins had a corresponding unigene and a total of 1,250 proteins were quantified and correlated. We identified the corresponding unigene for 36 of the 233 detected DAPs. The correlations were poor for the proteins and genes identified in the PFI-20 *vs.* PFI-60, PF *vs.* PFI-60, and PFI *vs.* PFI-20 (Table S14), suggesting the differences in transcript abundance may not be translated into changes at the protein level. This phenomenon may be due to transcription/post-transcription regulation, translation/post-translation regulation, protein modification, or protein–protein interactions.

We also compared the 36 PaWB-related proteins with the previous PaWB transcriptomics data. Because of the excessive number of assembled samples in these studies, we only compared the correlations between healthy and infected seedlings. We used BlastN to find matches for the unigenes analyzed in this study. We considered data from two *P. fortunei* transcriptome projects, PFa (Fan et al., 2015c) and PFb (Fan et al., 2014), two *P. tomentosa* transcriptome projects, PTa (Fan et al., 2015b) and PTb (Fan et al., 2015a), and a *P. tomentosa* × *P. fortunei* transcriptome project, PTF (Liu et al., 2013). Most of the proteins had corresponding unigenes, and a few of them matched DEUs (Table S15). Seven proteins detected in the present study, had corresponding DEUs in projects PFa and PFb, with six being common. This may be because all of the seedlings in these studies were *P. fortunei*. We identified nine, three, and seven proteins that had a corresponding DEU in the PTa, PFb, and PTF projects. Two, one, and three were common to the *P. fortunei* projects, respectively. This may be because of the different species and the hybridization that were used.

### Confirmation of DAPs by qRT-PCR

**Figure 4:**
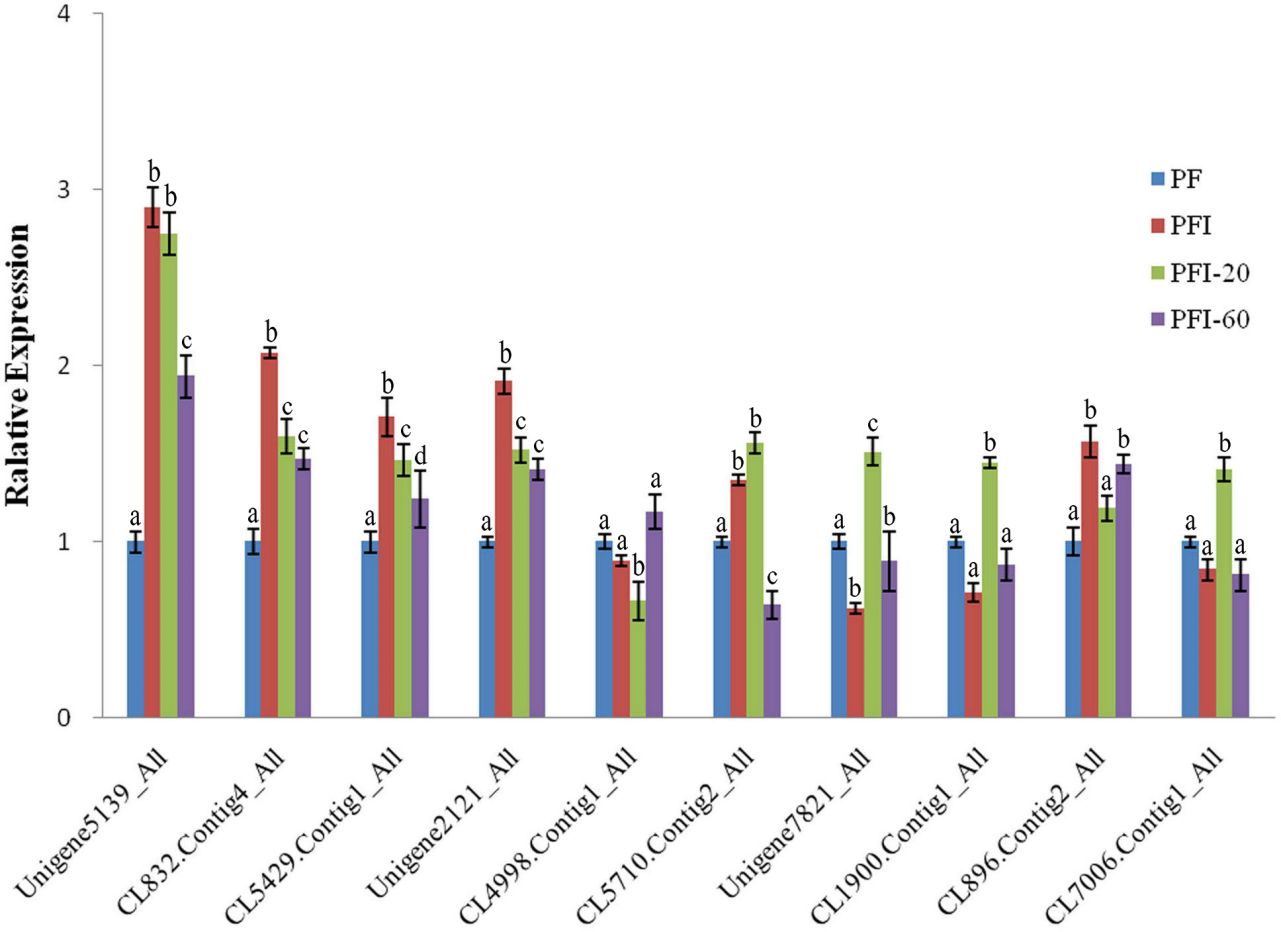
The expression of selected differentially abundant proteins at mRNA level. The 18S rRNA of Paulownia was chosen as an internal reference gene fornormalization. Unigene5139: GroES-like family protein, CL5429.Contig1: ribosomal protein L12, Unigene2121: MLP-like protein 423. CL4998.Contig1: rotamase cyclophilin 5, Unigene7821: Ribulose bisphosphate carboxylase (small chain) family protein, CL1900.Contig1: photosystem II oxygen-evolving enhancer protein 1, CL896.Contig2: HopW1-1-Interacting protein 1, CL7006.Contig1: FK506- and rapamycin-binding protein 15 KD-2, CL5710.Contig2: unknown protein, CL832.Contig4: photosystem I P700 chlorophyll a apoprotein A1. Standard error of the mean for three technical replicates is represented by the error bars. Different letters indicate significant differences.

To validate the DAPs identified by the iTRAQ analysis, we conducted qRT-PCR experiments to assess the expression of the genes encoding the DAPs at the mRNA level. Ten of the 36 PaWB-related proteins were randomly selected for validation, and primers were designed for the corresponding genes. The qRT-PCR results indicated that the transcript levels corresponding to eight of the DAPs were consistent with the iTRAQ results. The transcript levels for the other two DAPs and the corresponding protein abundances determined by iTRAQ exhibited the opposite trends (Fig. 4). This discrepancy may have been due to post-transcriptional and/or post-translational regulatory processes.

## Discussion

The analyses of genome-wide protein profiles induced by phytoplasmas is a powerful method for elucidating a plant’s responses to phytoplasma infections. In previous studies, the changes in transcription and the post-transcriptional regulatory activities upon PaWB phytoplasma infections were investigated. Based on the identified genes and miRNAs related to PaWB, researchers were able to produce a preliminary outline of the molecular mechanism associated with PaWB (Fan et al., 2015a; Fan et al., 2015b; Fan et al., 2014; Fan et al., 2016; Fan et al., 2015c; Liu et al., 2013). However, the results did not comprehensively reflect the changes in cell behavior directly, because most biological reactions involve proteins. Therefore, it was necessary to generate a Paulownia protein profile. In this study, we revealed the changes in protein abundance in *P. fortunei* seedlings upon phytoplasma infection using iTRAQ. We investigated the proteins profiles of healthy versus infected plants (PF *vs.* PFI) and recovered plants (PFI-20 *vs.* PFI-60), and found “photosynthesis” and “ribosome” metabolic pathways were both enriched among the DAPs in the two comparisons. The MMS-response proteins were identified as well, and some of them also mapped to “photosynthesis” and “ribosome” metabolic pathways. We further identified PaWB-related proteins through our comparison scheme. Finally, 36 PaWB-related proteins were obtained in this study; four of them were unknown proteins. The 32 proteins with known functions were divided into three groups: 11 proteins belonged to the carbohydrate and energy metabolism group (most were photosynthetic proteins); eight belonged to the protein synthesis and degradation group; and 13 were included in the stress resistance group. The proteins in the three groups exhibited the expected phytoplasma effects on photosynthesis and energy metabolism, amino acid and protein metabolism, and stress responses. Our data may help researchers clarify the pathogenesis of PaWB disease.

**Figure 5:**
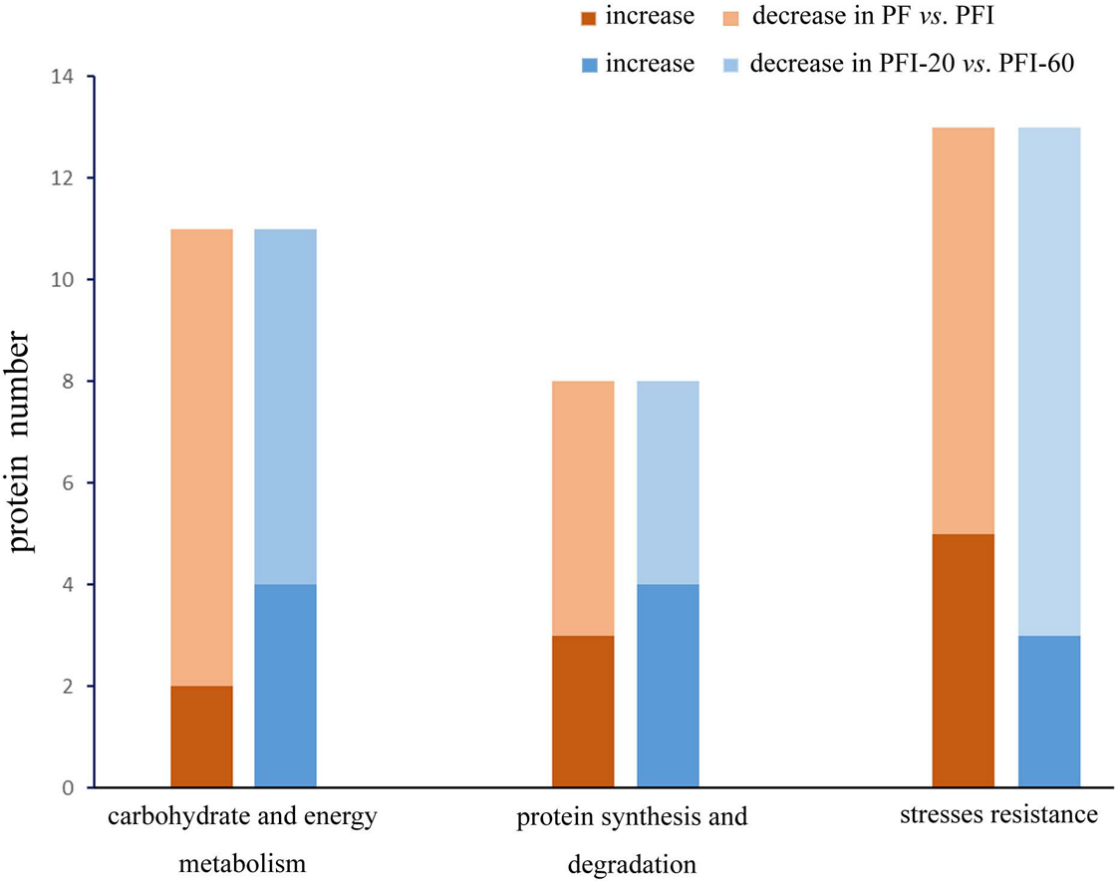
Proteins abundance change between PF *vs.* PFI and PFI-20 *vs.* PFI-60. Dark color: proteins abundance increase, light color: proteins abundance decrease.

To study the mechanisms regulating the PaWB disease and recovery processes, we investigated the 32 PaWB-related protein profiles in PF *vs.* PFI and PFI-20 *vs.* PFI-60 (Fig. 5). In particular, we focused on determining the changes in protein abundance during the progress from absence to presence of the PaWB-phytoplasma. In PF *vs.* PFI, the abundance of two and nine carbohydrate and energy metabolism proteins increased and decreased, respectively; in the protein synthesis and degradation group, the abundance of three and five proteins increased and decreased, respectively; and in the stress resistance group, the abundance of five and eight proteins increased and decreased, respectively. In PFI-20 *vs.* PFI-60, the abundance of four and seven carbohydrate and energy metabolism proteins increased and decreased, respectively; in the protein synthesis and degradation group, the abundance of four and four proteins increased and decreased, respectively; and in the stress resistance group, the abundance of three and ten proteins increased and decreased, respectively. In the two groups (carbohydrate and energy metabolism, protein synthesis and degradation), the number of proteins that belonged to the higher abundance and lower abundance categories decreased and increased, respectively. The variation trend in the stress resistance group was the opposite to that of the two groups. This might indicate a different response to PaWB among the three groups of proteins.

In the carbohydrate and energy metabolism group, compared with the accumulated proteins, the number of proteins that decreased in abundance in the infected process (PF *vs.* PFI) was large. This implies that carbohydrate and energy metabolism was impaired by the infection, which is consistent with previous results for lime (Monavarfeshani et al., 2013) and mulberry plants (Ji et al., 2009). In contrast, more proteins exhibited decreased abundance in the comparison of infected and recovered process. Therefore, we hypothesized that the presence of the PaWB-phytoplasma decreased the efficiency of carbohydrate and energy metabolism. After the phytoplasma was eliminated from the plants, the metabolic activities recovered to a certain extent. In the protein synthesis and degradation group, we observed that in the infected process, more proteins decreased abundance than that in the recovered process. After infection, the PaWB-phytoplasma may have disrupted protein synthesis and degradation, because phytoplasma are unable to synthetize amino acids. When the phytoplasma was removed, this dependence likely decreased.

In the stress resistance group, compared with the recovered process, more proteins exhibited decreased abundance rather than increased abundance in the infected process. After infection, the presence of PaWB-phytoplasma (in PFI) may induce stress resistance proteins to activate defense responses. In the recovered process, the PaWB-phytoplasma disappeared (in PFI-60), the number of proteins with increased abundance decreased, and the defense was not so robust. These reults may help to elucidate the stress resistance protein profile.

## Conclusions

In this study, comparative proteome-level analyses were performed for PF, PFI, PFI-20, and PFI-60 seedlings. Bioinformatics analyses of the identified proteins has provided the foundation of a protein database for further studies of PaWB disease in Paulownia. We investigated the DAPs in infected process (PF *vs.* PFI) and recovered process (PFI-20 *vs.* PFI-60), and found the “photosynthesis” and “ribosome” metabolic pathways might be important in the Paulownia–phytoplasma interaction. Through our comparison schemes, 36 PaWB-related DAPs, which were related to carbohydrate and energy metabolism, protein synthesis and degradation, and stress resistance were identified. The changes in the abundance of these proteins in PF *vs.* PFI and PFI-20 *vs.* PFI-60 were also investigated. We determined that the plant responses to the phytoplasma infection mainly involved three complementary categories of metabolic pathways. These results may enrich our understanding of plant–phytoplasma interactions, and will contribute to future investigations of the detailed mechanisms of Paulownia responses to phytoplasma infections.

##  Supplemental Information

10.7717/peerj.3495/supp-1 Figure S1 Detection of phytoplasma in Paulowina seedings ****1:PFI, 2:PFI-20, 3:PFI-60, 4:PFI-100, 5:PF, 6: ddH2O, D:DNA maker.

10.7717/peerj.3495/supp-2 Figure S2 The repeatability of two replicates (A) PF *vs.* PFI, (B) PF *vs.* PFI-60, (C) PFI-20* vs.* PFI-60, (D) PFI *vs.* PFI20. The ratios of protein abundances for each protein in each comparison between biological replicates were calculated, and the “delta, error” in the absciss are presents the difference from the expected ratio of 1.

10.7717/peerj.3495/supp-3 Figure S3 COG function classification of the identified proteins in *P. fortunei.* 1,489 proteins were divided into 23 specific categories.

10.7717/peerj.3495/supp-4 Figure S4 GO analyses of the identified proteins in *P. fortunei.* 2,139 proteins were categorized into 52 function groups.

10.7717/peerj.3495/supp-5 Figure S5 GO analysis of the DAPs in infected and recovered processes The light colors represent the significantly enriched GO terms. In infected processes, the number of all GO terms were 803, 139 and 184 for the three main GO categories, biological process, cellular component and molecular function. In recovered processes, the number were 679, 120 and 147 respectively.

10.7717/peerj.3495/supp-6 Table S1 Overview of the sequencing and assembly of the transcriptome of *P. fortunei.*

10.7717/peerj.3495/supp-7 Table S2 Primer sequence used in this study

10.7717/peerj.3495/supp-8 Table S3 Overview of the all identified proteins

10.7717/peerj.3495/supp-9 Table S4 KEGG annotation of the identified proteins

10.7717/peerj.3495/supp-10 Table S5 Overview of our comparison schemes comparisin 1:DAPs in PF *vs.* PFI comparisin 2: DAPs in PFI-20 *vs*. PFI60 comparison 3:non-DAPs in PF *vs.* PFI-60; comparison 4:non-DAPs in PFI *vs.* PFI-20; comparison 5: the common DAPs between PF *vs*. PFI and PFI-20 *vs*. PFI-60; comparison 6:uncommon proteins between cpmparison 3 and 4; comparison 7: common proteins between comparison 5 and 6.

10.7717/peerj.3495/supp-11 Table S6 Overview of our comparison schemes

10.7717/peerj.3495/supp-12 Table S7 GO analysis for the DAPs in PF* vs.* PFI

10.7717/peerj.3495/supp-13 Table S8 KEGG pathway analysis for the DAPs in PFI-20 *vs.* PFI-60

10.7717/peerj.3495/supp-14 Table S10 GO analysis for the DAPs in PFI-20* vs.* PFI-60

10.7717/peerj.3495/supp-15 Table S10 MMS-related DAPs

10.7717/peerj.3495/supp-16 Table S11 GO analysis of the DAPs related to PaWB

10.7717/peerj.3495/supp-17 Table S12 COG function classification of the DAPs related to PaWB

10.7717/peerj.3495/supp-18 Table S13 KEGG pathway analysis for the DAPs related to PaWB

10.7717/peerj.3495/supp-19 Table S14 Correlation analysis of transcriptome and proteome

10.7717/peerj.3495/supp-20 Table S15 Transcriptome change of the PaWB-realted proteins in previous study PFa : Fan et al. 491 2015c, PFb : Fan et al., 2014, PTa : Fan et al., 2015b , PTb : Fan et al., 2015a, PTF : Liu et al., 2013. Red: up-regulated; green: down-regulated.

## Additional Information and Declarations

### Data Availability

The following information was supplied regarding data availability: The sequencing data has been submitted to the Short Reads Archive under accession number SRP067302. Protein accession data is available via ProteomeXchange under project identifier PXD006731.
