# Supplementary figures and images for: Quantitative proteome-level analysis of paulownia witches’ broom disease with methyl methane sulfonate assistance reveals diverse metabolic changes during the infection and recovery processes
Source: PeerJ. 2017 Jul 3;5:e3495. doi: 10.7717/peerj.3495 (PMC5497676; doi:10.7717/peerj.3495)

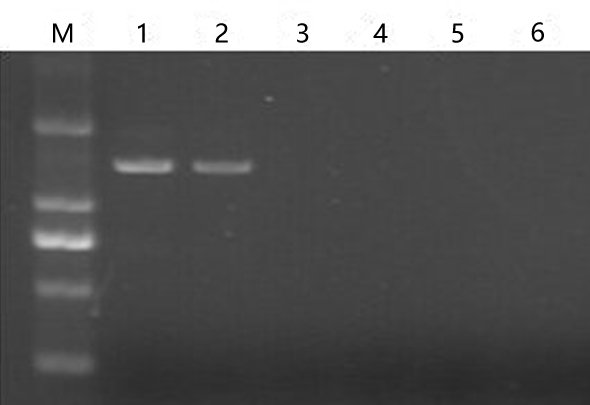

Supplement: Figure S1 — 1:PFI, 2:PFI-20, 3:PFI-60, 4:PFI-100, 5:PF, 6: ddH2O, D:DNA maker. [file peerj-05-3495-s001.jpg]

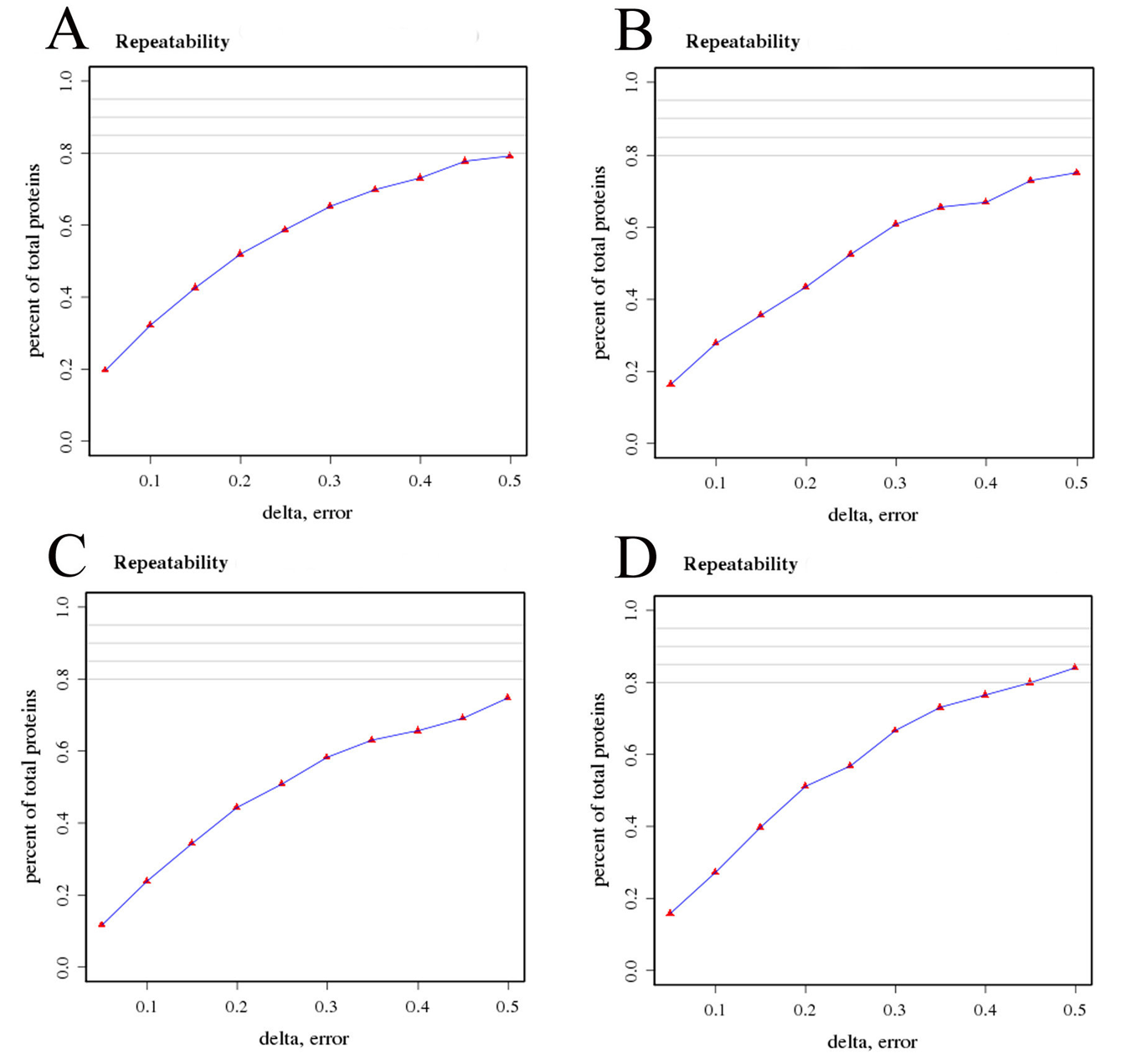

Supplement: Figure S2 — (A) PF vs. PFI, (B) PF vs. PFI-60, (C) PFI-20 vs. PFI-60, (D) PFI vs. PFI20. The ratios of protein abundances for each protein in each comparison between biological replicates were calculated, and the “delta, error” in the absciss are presents the difference from the expected ratio of 1. [file peerj-05-3495-s002.png]

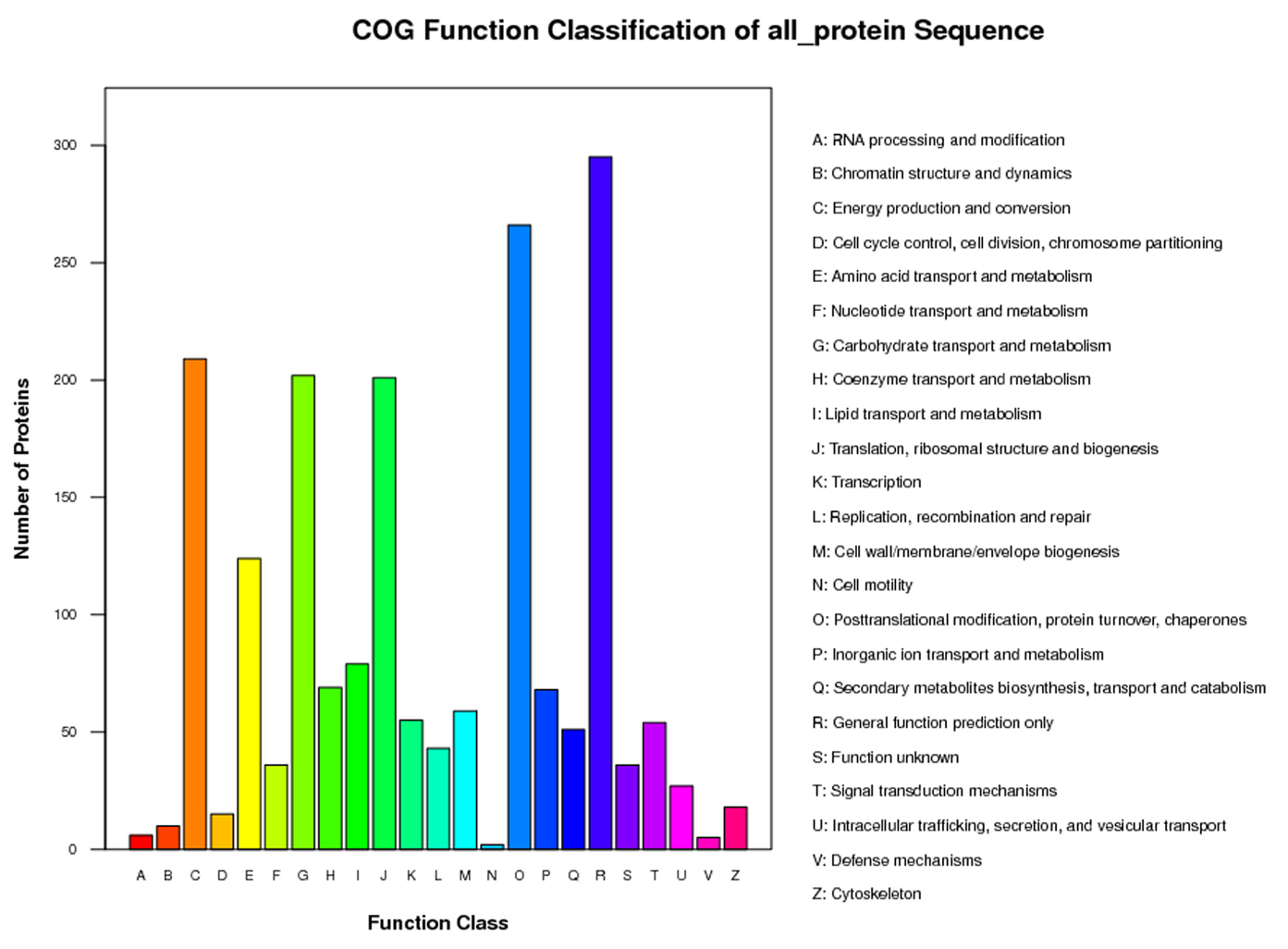

Supplement: Figure S3 — 1,489 proteins were divided into 23 specific categories. [file peerj-05-3495-s003.png]

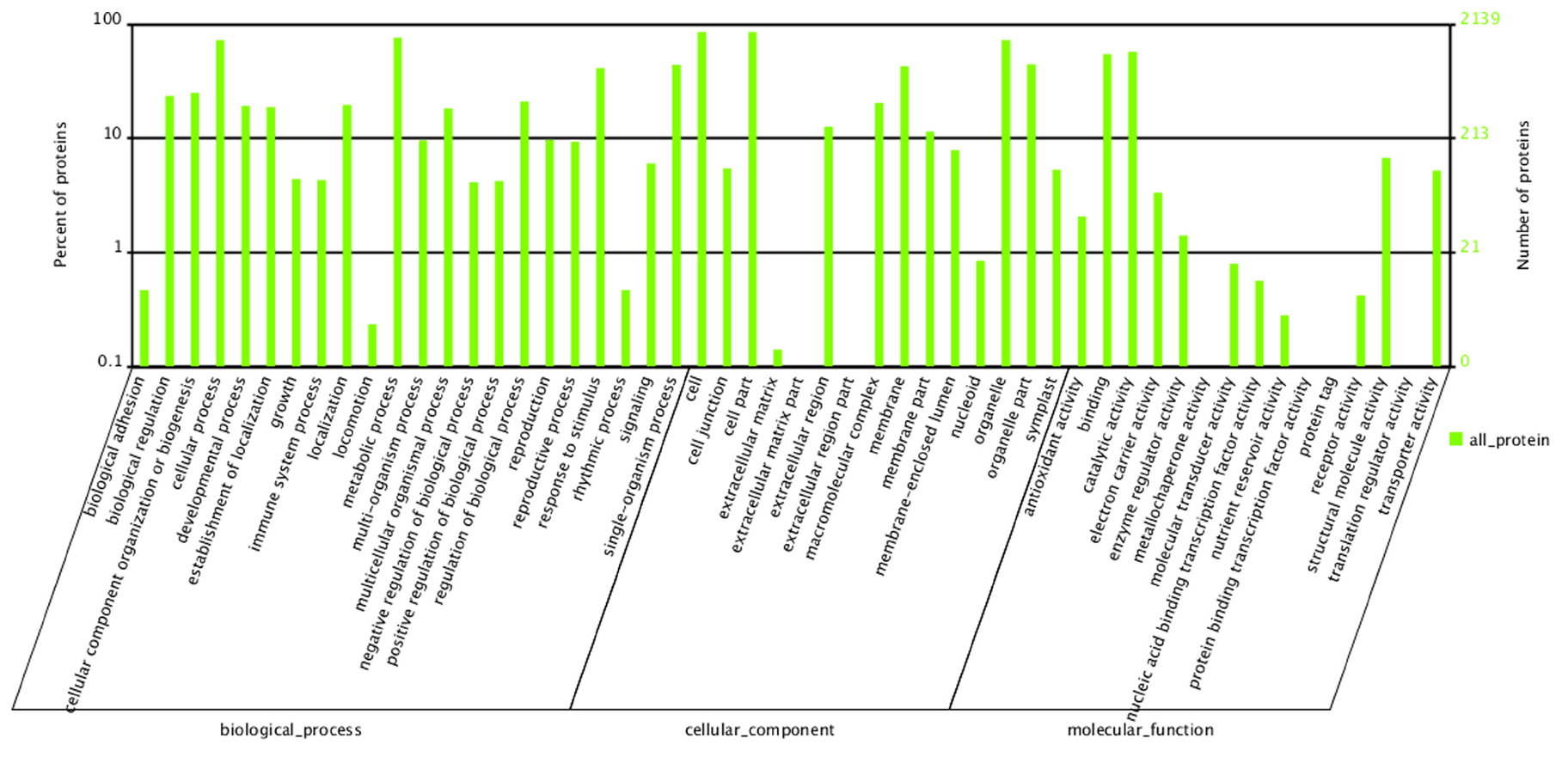

Supplement: Figure S4 — 2,139 proteins were categorized into 52 function groups. [file peerj-05-3495-s004.png]

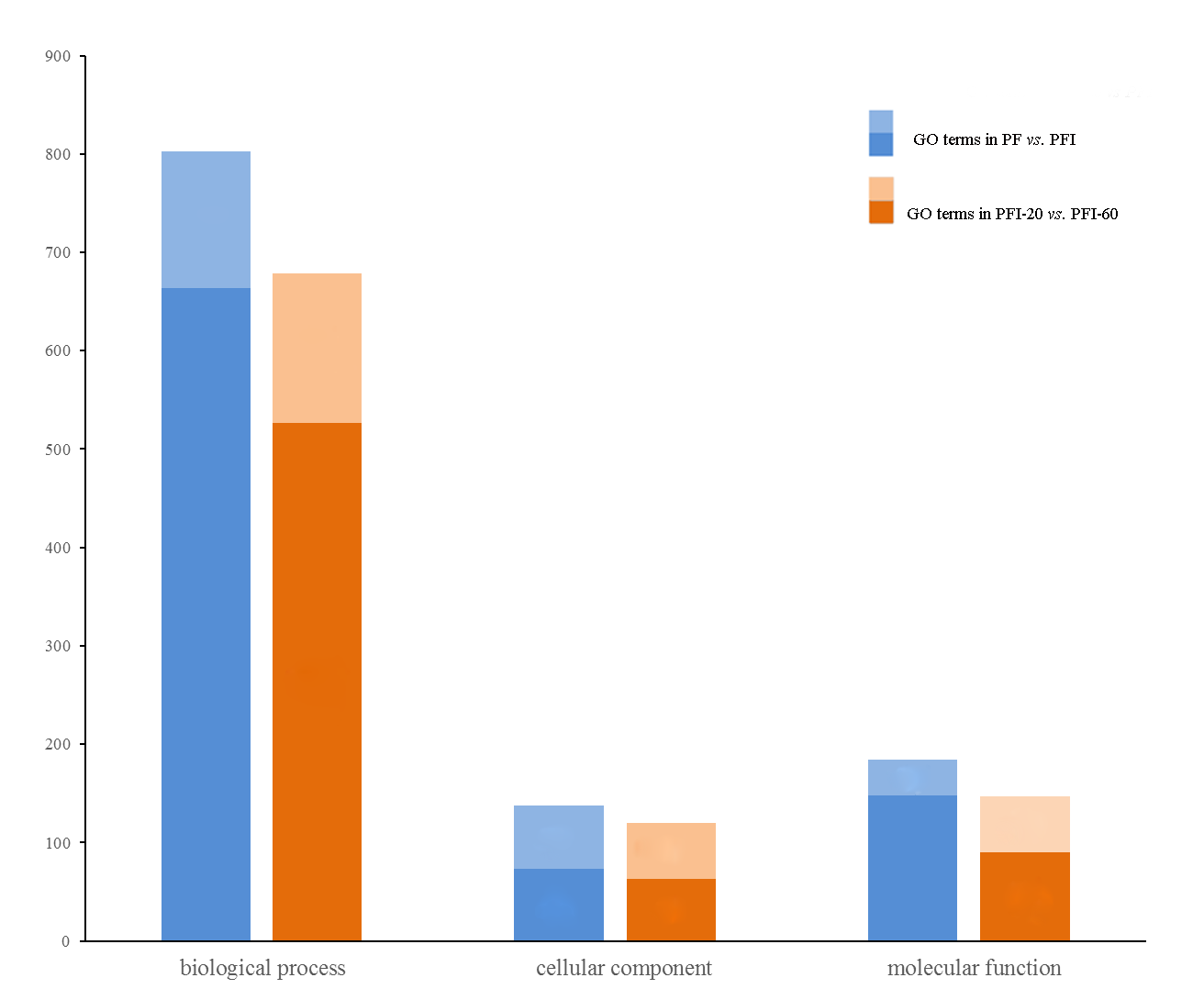

Supplement: Figure S5 — The light colors represent the significantly enriched GO terms. In infected processes, the number of all GO terms were 803, 139 and 184 for the three main GO categories, biological process, cellular component and molecular function. In recovered processes, the number were 679, 120 and 147 respectively. [file peerj-05-3495-s005.png]
